# Mortality in Adult Offspring of Immigrants: A Swedish National Cohort Study

**DOI:** 10.1371/journal.pone.0116999

**Published:** 2015-02-23

**Authors:** Hélio Manhica, Susanna Toivanen, Anders Hjern, Mikael Rostila

**Affiliations:** 1 Centre for Health Equity Studies, Stockholm University/Karolinska Institutet, Stockholm, Sweden; 2 Department of Clinical Epidemiology, Karolinska Institutet, Stockholm, Sweden; 3 Department of Sociology and Centre for Health Equity Studies, Stockholm University, Stockholm, Sweden; University of Bremen, GERMANY

## Abstract

**Background:**

Higher risks of psychiatric disorders and lower-than-average subjective health in adulthood have been demonstrated in offspring of immigrants in Sweden compared with offspring of native Swedes, and linked to relative socioeconomic disadvantage. The present study investigated mortality rates in relation to this inequity from a gender perspective.

**Methods:**

We used data from national registers covering the entire Swedish population aged 18-65 years. Offspring of foreign-born parents who were either Swedish born or had received residency in Sweden before school age (<7 years) were defined as “offspring of immigrants.” We used Cox regression models to examine the association between parental country of birth and mortality between 1990 and 2008, with adjustment for education, income, age and family type.

**Results:**

Male offspring of immigrants from the Middle East (HR:2.00, CI:1.66-2.26), other non-European countries (HR:1.80, CI:1.36-2.36) and Finland (HR:1.56, CI:1.48-1.65) showed an age-adjusted excess mortality risk from all causes of death when compared to offspring with Swedish-born parents. Income, but not education, greatly attenuated these increased mortality risks. No excess mortality rates were found among female offspring of immigrants, with the exception of external cause of death among offspring of Finnish immigrants.

**Conclusion:**

The study demonstrates high mortality rates in male offspring of immigrants from Finland and non-European countries that are associated with economic, but not educational, disadvantage. No increased mortality rates were found among female offspring of immigrants. Future studies are needed to explain this gender differential and why income, but not education, predicts mortality in male offspring of immigrants.

## INTRODUCTION

Immigrants in Sweden, as well as their offspring, have been found to have higher risks of various health problems: psychiatric morbidity, self-inflicted injuries [1], suicide [2], psychotic disorders [3], illicit drug abuse[4], as well as less favorable subjective health [5,6]. Studies have linked this higher burden of ill health to social inequity. Immigrants and their offspring have been singled out as a social category with difficult living conditions in Sweden, especially in the aftermath of the recession in the early 1990s [7], when unemployment rates increased more among immigrants and their offspring than among other segments of the population [8].

Some studies have suggested that female immigrants and offspring of immigrants are particularly vulnerable to health problems associated with the social disadvantage of migration [9,10]. There are both socioeconomic and behavioral explanations for potential health differentials by gender among offspring of foreign-born individuals. The situation of immigrant women is worse than that of men as regards gaining access to the labor market, and women have greater difficulty finding jobs that correspond to their qualifications [11,12]. Research into social inequalities in health shows that women are more vulnerable to developing depression when exposed to socioeconomic disadvantage [12,13]. Women also report less favorable subjective health in general when exposed to a low socioeconomic position [14]. Gender intersects with income, racial and ethnic hierarchies as well as with a number of other work-related social markers [10]. For instance, employment, access to the labor market and time in paid and unpaid work influence health differently in women than in men. With regard to behavioral risk factors, studies conducted in Sweden have shown that women smoke and drink less than men do, especially women from certain non-European countries [15]. Deaths from smoking-related diseases are more common among men than among women [16]. Health problems related to obesity are more common among non-European migrant women than among Swedish-born women [17,18]. Moreover, obesity may influence women’s and men’s labor market attachment differently, which may translate into lower earnings among women. Research shows that obese women have a higher risk of unemployment than do women of normal weight, yet similar results were not found among men [19].

### The socio-historical context of immigration in Sweden

Migration patterns to Sweden have changed character during the post-war period, and we might expect the composition of offspring with a foreign background to largely have followed these changes. Throughout the economic boom of the 1960s and 1970s, immigration to Sweden was dominated by a foreign labor force from the neighboring Nordic countries, in particular from Finland. However there was also labor immigration from other European countries such as Germany, Austria, Italy, Yugoslavia, Greece and Turkey. The global economic crisis in the 1970s together with changes in Swedish migration policy brought about changes in the immigration patterns as the demand for foreign labor declined significantly. Since that period, Sweden has experienced growing flows of asylum seekers and their families coming from non-European countries. In the 1970s, the majority of refugees came from Latin America. Throughout the 1980s, most refugees came from Iran and Iraq, and in the 1990s, Sweden also received a large number of refugees from the former Yugoslavia along with North Africa [6,20]. Experiences of discrimination, social exclusion and socioeconomic disadvantages have been considered common challenges faced by migrants in Sweden, especially among non-European migrants. A recent Swedish study shows that many specific groups are disadvantaged in terms of income and occupational status, despite their fairly high educational level [21]. However, whether a similar pattern is discernible among children of immigrants is not clear.

### Objectives

Although numerous studies have examined health and mortality among foreign-born individuals [21–23], data on the situation of their offspring are still limited. Thus, here we will study mortality among the offspring of foreign-born individuals in Sweden. Using unique longitudinal registry data covering the total Swedish population, we wished to investigate whether the socioeconomic inequity associated with having immigrant parents translates into mortality differentials and, if so, whether these patterns differ between men and women.

## METHODS

The present study is based on multiple-linked data from national Swedish routine registers including the total population register (TPR), National Population and Housing Censuses, the Longitudinal Data Base on Education, Income and Employment (LOUISE) and the Cause of Death register. The data are maintained at the Centre for Health Equity Studies (CHESS) in Stockholm.

### Ethics statements

The specific aims of the present study were approved by the Central Ethical Review Board of Stockholm (decision no. 2012/1260–31). All data used here were anonymous, and the researchers did not have access to any personal information that could identify study participants (e.g., personal identity number, home address, etc.). Consequently, it was not possible to trace specific individuals included in the dataset.

### Study population

The study population included all Swedish residents who were in the age range 18 to 65 during 1990–2008 according to the Register of the Total Population and who fulfilled the study’s parental country of birth criteria. The normal retirement age in Sweden is 65 years, and persons were excluded from the analysis when they turned 65 because we wished to study the influence of socioeconomic determinants of mortality among offspring of immigrants in the working age population. The cohort was followed for all-cause mortality and mortality due to external and natural causes by record linkage to the Cause of Death Register during 1990–2008. The study cohort consisted of 4,648,143 persons (see Table 1).

**Table 1:** Parents’ country/region of birth, person-time and number of deaths in the study population (1990–2008).

*Parents’ country of birth*		*N*	*Observations (Person/month)*	*Nr*. *Deaths*
Sweden	Sweden	4 451 115	72 126 798	142 217
Finland	Finland	93 629	1 492 945	1 744
Western countries	Germany, Belgium, France, Netherlands, Luxembourg, Norway, Island, Denmark, England, Italy, Switzerland, USA and other Western	27 918	442 780	497
Eastern Europe	Poland, Hungary, Bulgaria and other Eastern European countries	23 138	336 897	416
Former Yugoslavian republics	Yugoslavia, Slovenia, Croatia, Bosnia-Herzegovina, Macedonia	18 295	274 155	170
Other non-European	Africa, Latin America and South Asia	12 946	125 359	61
Middle East	Turkey, Iran, Iraq, Syria, Lebanon and other Middle East	21 102	212 231	124
All		4 648 143	75 011 165	145 228

We defined offspring of immigrants as individuals with two foreign-born parents who were either born in Sweden or had immigrated to Sweden before school age (0–6 years). Offspring of two Swedish-born parents were also included in the study population as a comparison group,[24] while offspring of two parents from different countries of birth were excluded from the study because we were less interested in children of mixed ethnic origin. Individuals who immigrated to Sweden before school age were studied together with Swedish-born children with two foreign-born parents, as previous Swedish studies have demonstrated that their health patterns are similar [3,22]. To ascertain that this held true in the present study as well, we performed an additional sensitivity analysis.

### Outcomes

We distinguished deaths from all causes, natural causes (ICD9 codes 0000–7969 or ICD10 codes A00-R99) and external causes of death (ICD9 codes 8000–9999 or ICD10 codes V01-Y98) using the Cause of Death Register. We used information on the year and month of death. For a description of the distribution of specific causes of death among groups of children of immigrants, see S1 Appendix.

### Co-variates

Gender: Indicates whether the person is a man or woman.

Age: Is a continuous variable and includes adults of working age, which is between the ages of 18 and 65, at any time during the follow-up period.

Education: Is classified into three levels: “Primary” consists of compulsory school and pre-secondary education of less than 9 years, “secondary” covers three years of upper secondary school and “university” comprises tertiary education at university colleges.

Family type: Is divided into 3 levels: “other family status”, “married/cohabiting with at least one child, and “single parents”.

Disposable income: This variable is based on information on the individual part of the household’s annual disposable income and comprises various sources of income after tax. The variable is equally divided into 5 quintiles: Quintile 1 compromises those whose income is in the lowest 20% of the total income range, while quintile 5 corresponds to those whose income is in the highest 20%.”

### Statistical analysis

The analysis was based on person-time measured from January 1990 to December 2008.

Person-time was measured from January 1990 or the year the individual turned 18, until December 2008 or when he/she died, emigrated or turned 65.

Cox proportional hazards of gender-stratified models were estimated to contrast all-cause, as well as cause-specific, mortality rates among individuals by parents’ ‘country of birth’ categories. All co-variates were introduced as time-dependent variables on a yearly basis in the models. All models were adjusted for age. Model 2 was also adjusted for income only, Model 3 also for education only, and the final model included all aforementioned variables and family type (Model 4). The proportional hazards assumption was formally assessed using a Grambsch-Therneau test performed on the basis of the Schoenfeld residuals from each Cox regression model [25]

## RESULTS

Table 1 presents the study population by parents’ country/region of birth. The largest group of immigrant offspring was found among Finns, with 93,629 individuals born in Sweden or having immigrated to Sweden under the age of 7, with both parents born in Finland. The smallest group was those with both parents born in Africa, Latin America and South Asia, accounting for a total of 12,946 individuals. A total of 75,011,165 person-months were analyzed, and there were 145,228 deaths during the follow-up period. The distribution of the most common causes of death by parent’s country of birth is presented in S1 Appendix. Table 2 describes the socioeconomic indicators of the study population. The table reveals that offspring of immigrants from the Middle East and the former Yugoslavian republics had the lowest mean age compared to other immigrant offspring. The table also shows that there are no major educational differences within the study population. On the other hand, we found a considerable difference in distribution of income across the study population. In fact, descendants of immigrants from the Middle East and other non-European countries are overrepresented among those with the lowest total income. When socioeconomic conditions (i.e., education and income) were stratified by gender (see S2 Appendix), the results indicated that the proportion with a university-level education was somewhat higher among women than among men. We also found higher percentages of men with a primary education compared to women. Despite higher education, women descendants of immigrants had a lower income than their male counterparts did.

**Table 2:** Distribution of socioeconomic indicators across countries presented as proportions (%).

*Variable name*	*Sweden*	*Western*	*Finland*	*Yugoslavia republics*	*Other non-European*	*Middle East*	*Eastern Europe*
Gender							
Male	51.07	51.57	50.92	52.23	51.38	51.89	51.89
Female	48.93	48.43	49.08	47.77	48.62	48.11	48.11
Income							
1quintile	16.77	16.93	19.17	32.54	61.04	60.92	30.83
2quintile	20.18	19.00	22.06	21.83	18.96	18.63	17.30
3quintile	20.97	20.65	22.09	18.65	9.96	10.25	15.74
4quintile	21.09	21.80	21.71	16.46	6.41	6.63	16.03
5quintile	20.99	21.63	14.97	10.52	3.63	3.84	20.10
Education							
Primary	21.07	21.36	20.28	16.84	26.97	31.97	18.75
Secondary	64.66	66.72	71.67	74.49	64.17	61.36	62.58
University	14.26	11.89	8.05	8.67	8.86	6.67	18.67
Mean age	40	37	33	28	24	24	35

Table 3 shows a gender-stratified Cox regression for all-cause mortality. Model 1 shows that male offspring of immigrants from the Middle East (HR: 2.00, 95% CI: 1.66–2.46), other non-European countries (HR: 1.80, 95% CI: 1.36–2.36) and Finland (HR: 1.56, 95% CI: 1.48–1.65) experienced higher mortality risks than Swedish males did. When income was adjusted for in Model 2, the mortality risks were attenuated considerably in all groups except among Finnish offspring of immigrants (HR: 1.53, 95% CI: 1.45–1.62). In contrast, there was only marginal attenuation of these mortality risks when education, but not income, was adjusted for in Model 3. Finally, in Model 4 (fully adjusted for family type, education, age and income), patterns were similar to the income-only adjusted model (Model 2).

**Table 3:** Hazard ratios (HR) of all-cause mortality in offspring of immigrants in Sweden, 1990–2008.

*Parents’ country/region of birth*	*HR (Age)*		*HR (Age and Income)*		*HR (Age and Education)*		*HR (Age*, *Income*, *Education*, *Family type)*		*Death*
Men	Model1	[95% C.I]	Model2	[95% C.I]	Model3	[95% C.I]	Model4	[95% C.I]	N
Sweden	1.00		1.00		1.00		1.00		88 411
Finland	1.56	1.48–1.65	1.53	1.45–1.62	1.64	1.55–1.74	1.52	1.44–1.61	1 261
Western countries	0.90	0.81–1.01	0.93	0.83–1.03	0.93	0.83–1.04	0.92	0.82–1.03	319
Eastern Europe	1.00	0.88–1.12	0.97	0.86–1.10	1.02	0.90–1.15	0.99	0.87–1.11	265
Former Yugoslavian republics	1.39	1.17–1.65	1.14	0.95–1.35	1.43	1.20–1.70	1.18	1.00–1.41	130
Middle East	2.00	1.66–2.46	1.12	1.01–1.50	2.01	1.71–2.56	1.32	1.08–1.62	99
Other non-European	1.80	1.36–2.36	1.16	0.88–1.53	1.93	1.45–2.56	1.25	0.94–1.67	51
Women									
Sweden	1.00		1.00		1.00		1.00		53 806
Finland	1.01	0.98–1.17	1.12	1.02–1.23	1.12	1.02–1.23	1.13	1.03–1.23	483
Western countries	0.91	0.78–1.05	0.97	0.83–1.12	0.96	0.82–1.10	0.89	0.84–1.13	178
Eastern Europe	0.99	0.84–1.22	1.04	0.88–1.22	1.03	0.88–1.21	1.05	0.90–1.24	151
Former Yugoslavian republics	0.87	0.63–1.18	0.82	0.60–1.12	0.92	0.67–1.26	0.85	0.62–1.16	40
Middle East	1.11	0.75–1.28	0.87	0.58–1.28	1.13	0.75–1.71	0.86	0.57–1.30	25
Other non-European	0.75	0.40–1.41	0.63	0.34–1.17	0.91	0.48–1.68	0.70	0.37–1.29	10

Unadjusted mortality patterns among female offspring of immigrants in Table 3 reveal no excessive mortality risks. In female offspring of Finnish immigrants, however, a slightly increased risk is present in the adjusted models (Model 2–4). For all other female offspring of immigrants, the fully adjusted models generally show no or even lower mortality risks compared with offspring of native Swedes, although the results are not statistically significant.

In Table 4, mortality risks are presented separately for external and natural causes for men only. Mortality rates and attenuation after adjustment for income were similar for these two broad causes of death, as compared to deaths from all causes. For offspring of Finnish women, there was a notable difference, with a crude increased HR in external causes of death 1.47 (1.27–1.72), which increased further to 1.65 (1.45–1.90) with adjustment for income, while there were no increased mortality risks for natural causes. These results are not shown in the tables, as they were not significant for all other offspring of immigrants.

**Table 4:** Hazard ratios (HR) of mortality from external and natural causes in offspring of immigrants in Sweden, 1990–2008 (MEN).

*Parents’ country/region of birth*	*HR (Age)*		*HR (Age and Income)*		*HR (Age and Education)*		*HR (Age*, *Income*, *Education*, *Family type)*		*Death*
External causes of death	Model1	[95% C.I]	Model2	[95% C.I]	Model3	[95% C.I]	Model4	[95% C.I]	N
Sweden	1.00		1.00		1.00		1.00		18 887
Finland	1.73	1.59–1.89	1.57	1.45–1.70	1.84	1.70–2.00	1.37	1.27–1.48	606
Western countries	0.88	0.72–1.08	0.84	0.69–1.02	0.96	0.79–1.17	0.96	0.84–1.09	99
Eastern Europe	1.05	0.85–1.29	0.92	0.74–1.13	1.15	0.93–1.41	0.98	0.84–1.13	88
Former Yugoslavian republics	1.27	1.01–1.58	1.07	0.86–1.34	1.33	1.07–1.67	1.02	0.77–1.34	78
Other non-European	1.39	1.00–1.92	0.96	0.69–1.34	1.50	1.08–2.09	0.82	0.47–1.42	36
Middle East	1.53	1.21–1.94	1.07	0.84–1.37	1.15	1.21–1.96	0.94	0.65–1.36	68
Natural causes of death									
Sweden	1.00		1.00		1.00		1.00		69524
Finland	1.42	1.32–1.54	1.26	1.20–1.40	1.49	1.88–2.22	1.78	1.83–2.18	655
Western countries	0.95	0.83–1.08	0.92	0.81–1.06	0.98	0.79–1.19	0.96	0.80–1.20	220
Eastern Europe	0.96	0.84–1.12	0.95	0.81–1.01	1.00	0.50–1.12	1.05	0.50–1.12	177
Former Yugoslavian republics	1.23	0.93–1.62	0.99	0.75–1.29	1.25	0.89–1.39	1.18	0.87–1.38	52
Other non-European	1.36	0.82–2.25	0.86	0.52–1.43	1.29	1.02–2.12	1.07	0.94–2 02	15
Middle East	1.64	1.15–2.34	1.05	0.73–1.50	1.52	0.79–1.24	1.08	0.75–1.18	31

A sensitivity analysis was performed, where the crude estimates for Swedish-born offspring of immigrants only were compared with those including children who came to Sweden before age 7. The results (not shown in the table) indicated that male offspring of immigrants from the Middle East (HR: 1.78, CI: 1.29–2.38), other non-European countries (HR: 1.82, CI: 1.16–2.86), former Yugoslavian republics (HR: 1.19, CI: 1.23–1.73) and Finland (HR: 1.76, CI: 1.64–1.89) experienced higher mortality risks than Swedish males did. The estimated results decreased considerably when income was adjusted for, except among offspring of Finnish immigrants: HR = 1.69 (1.58–1.82). No excess risk mortality patterns were found among female offspring of immigrants. To conclude, including offspring who arrived before school age into our analyses did not alter the overall findings.

## DISCUSSION

The results of the present study on a national cohort of adult offspring of immigrants in Sweden demonstrate twofold increased mortality among offspring of non-European immigrants and more modestly increased mortality rates among offspring of immigrants from Finland and the former Yugoslavia compared with the majority population. These increased risks were greatly attenuated after adjustment for income, but not for education. For female offspring no increased risks were found, with the exception of increased mortality in external cause death for offspring of Finnish immigrants, which was not attenuated either by income or by education.

One major finding in the present study was that income, but not education, attenuated the mortality differentials between male offspring of immigrants compared with the majority population. This effect was particularly strong among the offspring of non-European immigrants, where mortality rates were the highest. Offspring of non-European immigrants are easy to identify in a Swedish context, owing to their physical appearance and non-Swedish names. Studies in Canada [26], Sweden [23,27], the US [28] and Germany [29] have also shown that native Blacks and other offspring of non-European immigrants experience significant disadvantages in earnings as a consequence of discrimination on the employment market and within certain occupational sectors. Thus, discrimination on the labor market, which results in education not paying off in the same way as it does in the majority population, is congruent with the income-education pattern found here. Numerous studies [30,31] have suggested that perceived discrimination is a chronic stressor that leads to ill health, and we may speculate that chronic stress of this kind is also important in understanding the present findings of higher mortality rates among Swedish residents of non-European origin. Accordingly, it has been argued that occupational mismatch per se is associated with poor mental health, for instance through feelings of shame [32–34]. This may especially influence the health of overeducated workers with an immigrant background, who are more likely to experience occupational mismatch and its potential consequences in the form of psychological stress and shame [33]. Further studies are needed to confirm this hypothesis.

The present study confirms a large body of previous studies describing an excess risk of ill health among Swedish residents of Finnish origin [2,35–39]. Alcohol abuse and high smoking rates have been implicated as important risk factors for this increased morbidity and mortality in the Finnish population in Sweden [40,41]. Our findings also suggest that higher mortality rates among offspring of Finnish immigrants are in accord with the mortality risk of their foreign-born parents [21].

The higher mortality risks of male offspring of non-European immigrants are in stark contrast to the lower mortality rates observed in their parents in a previous Swedish study[37]. Considering that first-generation non-European immigrants have been found to have considerably lower incomes compared with their offspring, socioeconomic factors cannot be expected to be important in explaining this differential. A healthy migrant hypothesis [42], on the other hand, would fit this pattern, assuming that individuals with good health in the country of origin were more prone to migrate to Sweden, while their offspring were not selected in this manner. Accordingly, previous evidence from the US suggests that the offspring of immigrants experience poorer health than their foreign-born parents do and that second-generation migrants have adopted a mortality pattern equivalent to that of the native population [43].

The present study did not confirm hypotheses about female vulnerability to the health consequences of socioeconomic disadvantage associated with migration. On the contrary, men with non-European parents were found to have the highest mortality rates for external as well as natural causes. In general, scholars have argued that women are more vulnerable to socioeconomic disadvantage than men are and that this is a consequence of gender discrimination, unemployment and conflicting gender role expectations [44,45]. Nevertheless, our findings indicated that men were more likely than women to experience higher mortality risks. It is plausible that the excess mortality risks faced particularly by Finnish as well as non-European offspring could be explained by disparities in gender differences in behavioral factors related to one socioeconomic position [46]. Smoking is one risk factor that could fit such a pattern, where men with a Middle Eastern heritage have been found to have particularly high rates, while women with a Middle Eastern heritage have been shown to have lower rates compared to the majority population [5]. Use and abuse of illicit drugs is another factor that shows a similar pattern [4]. To a certain extent, men and women compete on different labor markets, which could be another factor of interest in explaining these gender differences [47].

### Methodological limitations

Besides the obvious strengths of our study, such as the use of population register data, the extent of the study period and longitudinal follow-up, there are some methodological concerns and limitations that should be raised. The concept “offspring of immigrants” could be questioned, as individuals who immigrated under the age of 7 years were included in this group. We do believe, however, that children who immigrate before school age have more in common with children born after resettlement of the family than do adult or teenage immigrants, and this is corroborated by studies of educational achievement in the US [48]. This assumption was supported by the sensitivity test reported above.

Furthermore, we restricted our study population to children of immigrants in the working age population, i.e. 18–65 years. We decided not to include old-age pensioners, because they may have health issues that are different from those found in the working population. Including persons over the age of 65 years would also involve difficulties in interpreting the contribution of confounders and especially income to the association of primary interest, because income would have been related to the retirement pension system rather than to current employment. Finally, within this restricted population of children of immigrants, the majority of whom arrived in Sweden after the 1950s and 1960s, very few individuals are older than 65 years. Data on age distribution showed that the proportion of children of immigrants above the age of 65 is very low (less than 1%). Yet this age restriction means that we can only generalize our results to the working age population and not to people of retirement age.

One general shortcoming of register data is that essential information about the mechanisms underlying associations is lacking. For instance, the data used in our study did not contain information related to health behavior, social support or measurement of different types of discrimination related to gender and ethnic background that could account for the effect of individuals’ sociodemographic characteristics on the observed gender and ethnic mortality pattern. Thus, our study needs to be complemented by other studies that include such variables.

### Conclusion

The present study demonstrates increased mortality risks among adult male offspring of immigrants from non-European countries in Sweden, and a more modest increase among offspring of immigrants from the former Yugoslavia and Finland. This increased mortality in male offspring of immigrants was greatly attenuated by adjustment for income, but not education. Female offspring of immigrants, in contrast, had similar or even lower mortality rates compared with the majority population, with the exception of female offspring of immigrants from Finland. Further studies, with more detailed data on work and employment history, are needed to elucidate the mechanisms underlying these striking differentials in mortality among the offspring of immigrants.

## Supporting Information

S1 Appendix Distribution of the common cause of death presented as proportions (%). (DOCX)

S2 Appendix Distribution of income and education by gender presented as proportions (%). (DOCX)
