# Supplementary material for: Mortality in Adult Offspring of Immigrants: A Swedish National Cohort Study
Source: PLoS One. 2015 Feb 23;10(2):e0116999. doi: 10.1371/journal.pone.0116999 (PMC4338186; doi:10.1371/journal.pone.0116999)
Supplement: S1 Appendix — (DOCX) [file pone.0116999.s001.docx]

Appendix S1. Distribution of the common cause of death presented as proportions (%)

|  | Natural causes of death | | | | | |  | External causes of death | | |
| --- | --- | --- | --- | --- | --- | --- | --- | --- | --- | --- |
| Parents’ country/region of birth | Infectious diseases | Cancer | Circulatory diseases | Respiratory diseases | Disease of nervous system | Other Natural |  | Accident | Suicide | Injury and other external cause of death |
| Sweden | 1.25 | 49.08 | 28.16 | 4.03 | 3.52 | 13.96 |  | 58.73 | 27.56 | 13.71 |
| Western countries | 2.64 | 36.50 | 23.84 | 3.27 | 5.38 | 21.13 |  | 57.48 | 29.92 | 12.6 |
| Finland | 2.32 | 45.62 | 23.71 | 3.87 | 3.35 | 28.38 |  | 57.11 | 25.97 | 16.93 |
| Former Yugoslavia | 3.70 | 33.33 | 18.52 | 14.81 | 14.81 | 35.53 |  | 40.63 | 28.13 | 31.25 |
| Other non-European | 1.56 | 35.94 | 20.31 | 3.13 | 10.94 | 14.81 |  | 41.46 | 43.9 | 14.63 |
| Middle East | 0.95 | 46.03 | 26.03 | 2.86 | 4.76 | 28.13 |  | 40 | 31.25 | 28.75 |
| Eastern Europe | 5.26 | 30.26 | 14.47 | 2.63 | 11.84 | 19.37 |  | 55 | 26.67 | 18.33 |
