# Supplementary material for: Mortality in Adult Offspring of Immigrants: A Swedish National Cohort Study
Source: PLoS One. 2015 Feb 23;10(2):e0116999. doi: 10.1371/journal.pone.0116999 (PMC4338186; doi:10.1371/journal.pone.0116999)
Supplement: S2 Appendix — (DOCX) [file pone.0116999.s002.docx]

Appendix S 2. Distribution of income and education by gender presented as proportions (%)

| Variable name | Sweden | | Western | | Finland | | Former Yugoslavia | | Other non-European | | Middle East | | | Eastern Europe | |
| --- | --- | --- | --- | --- | --- | --- | --- | --- | --- | --- | --- | --- | --- | --- | --- |
| Gender | Male | Female | Male | Female | Male | Female | Male | Female | Male | Female | Male | Female | Male | | Female |
| Income |  |  |  |  |  |  |  |  |  |  |  |  |  | |  |
| **1quintile** | 14.86 | 18.77 | 16.91 | 17.55 | 19.57 | 18.76 | 33.33 | 31.67 | 61.86 | 60.16 | 62.02 | 59.51 | 30.33 | | 31.37 |
| **2quintile** | 13.96 | 26.68 | 14.15 | 24.25 | 17.22 | 27.10 | 18.18 | 25.85 | 16.87 | 21.43 | 15.65 | 21.31 | 13.99 | | 20.90 |
| **3quintile** | 17.81 | 24.27 | 16.95 | 24.64 | 18.89 | 25.43 | 16.57 | 20.95 | 9.57 | 10.41 | 9.54 | 11.03 | 12.92 | | 18.82 |
| **4quintile** | 24.19 | 17.85 | 23.85 | 19.58 | 24.74 | 18.55 | 18.32 | 14.42 | 7.17 | 5.59 | 7.63 | 5.54 | 16.64 | | 15.36 |
| **5quintile** | 29.18 | 12.43 | 28.70 | 13.99 | 19.59 | 10.16 | 13.60 | 7.12 | 4.77 | 2.40 | 5.15 | 2.40 | 26.12 | | 13.55 |
| Education |  |  |  |  |  |  |  |  |  |  |  |  |  | |  |
| **Primary** | 23.33 | 18.72 | 22.20 | 20.52 | 21.42 | 19.09 | 17.94 | 15.63 | 29.31 | 24.45 | 37.04 | 26.45 | 19.46 | | 17.97 |
| **Secondary** | 63.53 | 65.84 | 66.55 | 66.91 | 72.85 | 71.26 | 74.85 | 74.10 | 63.31 | 64.73 | 57.58 | 65.48 | 62.33 | | 62.86 |
| **University** | 13.14 | 15.43 | 11.25 | 12.57 | 6.51 | 9.65 | 7.21 | 10.27 | 7.05 | 10.81 | 5.39 | 8.07 | 18.22 | | 19.16 |
